# The origins of light-independent magnetoreception in humans

**DOI:** 10.3389/fnhum.2024.1482872

**Published:** 2024-11-29

**Authors:** Takashi Shibata, Noriaki Hattori, Hisao Nishijo, Satoshi Kuroda, Kaoru Takakusaki

**Affiliations:** ^1^Department of Neurosurgery, Toyama University Hospital, Toyama, Japan; ^2^Department of Neurosurgery, Toyama Nishi General Hospital, Toyama, Japan; ^3^Department of Rehabilitation, Toyama University Hospital, Toyama, Japan; ^4^Faculty of Human Sciences, University of East Asia, Yamaguchi, Japan; ^5^The Research Center for Brain Function and Medical Engineering, Asahikawa Medical University, Asahikawa, Japan

**Keywords:** geomagnetic field, magnetoreception, magnetotactic bacteria, electromagnetic induction, semicircular canals, iron

## Abstract

The Earth’s abundance of iron has played a crucial role in both generating its geomagnetic field and contributing to the development of early life. In ancient oceans, iron ions, particularly around deep-sea hydrothermal vents, might have catalyzed the formation of macromolecules, leading to the emergence of life and the Last Universal Common Ancestor. Iron continued to influence catalysis, metabolism, and molecular evolution, resulting in the creation of magnetosome gene clusters in magnetotactic bacteria, which enabled these unicellular organisms to detect geomagnetic field. Although humans lack a clearly identified organ for geomagnetic sensing, many life forms have adapted to geomagnetic field—even in deep-sea environments—through mechanisms beyond the conventional five senses. Research indicates that zebrafish hindbrains are sensitive to magnetic fields, the semicircular canals of pigeons respond to weak potential changes through electromagnetic induction, and human brainwaves respond to magnetic fields in darkness. This suggests that the trigeminal brainstem nucleus and vestibular nuclei, which integrate multimodal magnetic information, might play a role in geomagnetic processing. From iron-based metabolic systems to magnetic sensing in neurons, the evolution of life reflects ongoing adaptation to geomagnetic field. However, since magnetite-activated, torque-based ion channels within cell membranes have not yet been identified, specialized sensory structures like the semicircular canals might still be necessary for detecting geomagnetic orientation. This mini-review explores the evolution of life from Earth’s formation to light-independent human magnetoreception, examining both the magnetite hypothesis and the electromagnetic induction hypothesis as potential mechanisms for human geomagnetic detection.

## Introduction

The Earth, an iron-rich planet, functions as a massive magnet with a North and South Pole, with iron playing a crucial role in driving evolution (Wade et al., 2021). Additionally, iron is essential for the synthesis and repair of Deoxyriboucleic acid (DNA) and is believed to have functioned as a catalyst for the origin of life (Puig et al., 2017). Subsequently, organisms acquired the ability to biomineralize, or produce magnetite (Fe3O4), within their bodies. For example, chitons use this ability to develop the strongest teeth capable of biting rock (Nemoto et al., 2019), and magnetotactic bacteria (MTB) evolved to move in response to the geomagnetic field (Goswami et al., 2022; Strbak et al., 2022).

In the geomagnetic navigation of animals, there is not only a compass response indicating magnetic north and south (Lohmann et al., 2001) but also a magnetic map involving more complex information processing that incorporates various elements such as the intensity and direction of the geomagnetic field (Wiltschko and Wiltschko, 2005). Migratory birds are sensitive to geomagnetic field from a young age. As they grow, they develop the ability to accurately determine the direction of geomagnetic field, enabling them to migrate according to the seasons (Wiltschko and Wiltschko, 1972). Besides migratory birds, many animals including insects possess the sense of magnetoreception (Wiltschko and Wiltschko, 2012), allowing them to detect geomagnetic field. Notably, desert ants have enhanced their magnetic compass abilities to adapt to extreme high-temperature environments (Fleischmann et al., 2020). Additionally, while the magnetized abdomen of honeybees is thought to be a magnetoreceptive organ (Liang et al., 2016), it has also been suggested that it functions to store iron waste from their food (Shaw et al., 2018). This has drawn attention to the antennae of honeybees, which function as multimodal mechanoreceptive sensory organs and might also contain magnetite (Serna et al., 2024). The Johnston’s organ in the antennae, which can detect minute air vibrations and weak gravitational inclines, is also suggested to be a potential magnetoreceptor (Fleischmann et al., 2020). However, behavioral studies alone are insufficient to conclude magnetoreception in animals (Schneider et al., 2023). Therefore, it is necessary to elucidate the clear physiological mechanisms for detecting geomagnetic field in humans, including light-dependent pathways involving cryptochromes and light-independent pathways involving magnetite or electromagnetic induction.

Modern humans have long been thought to have lost the ability to sense geomagnetic field. Interestingly, even in dark environments, it has been found that the human brain’s alpha rhythms respond to fluctuating magnetic fields of similar intensity to the Earth’s natural direction (Wang et al., 2019). However, this magnetic perception in humans is much weaker than vision or hearing and is not consciously perceived. This study suggested the presence of a light-independent transduction pathway involving magnetite, but the sensory organs for this sense have not been identified, leaving human magnetoreception a deep mystery. To solve this difficult problem, it is necessary to consider the evolutionary history of life, where iron molecules have adaptively evolved to the geomagnetic field since the birth of life.

Extensive research on magnetoreception in vertebrates, including fish, birds, and mammals, suggests that modern humans might possess two potential transduction pathways: a light-dependent pathway through the optic nerve (Chae et al., 2022) and a light-independent pathway through the trigeminal and vestibular nerves (Wang et al., 2019). The light-dependent pathway likely emerged during the Cambrian explosion, alongside the evolution of eyes (Parker, 2005). In contrast, the light-independent pathway is thought to originate from MTB and the magnetosome gene cluster (Bellinger et al., 2022). Given its deep connection to iron-based metabolic systems, this pathway might trace back to the origins of Earth and life itself. Additionally, the semicircular canals of vertebrates, which are sensory organs that detect gravity and head rotation (Higuchi et al., 2019), might have evolved to sense geomagnetic field through electromagnetic induction (Nimpf et al., 2019).

The activation of zebrafish and medaka hindbrain neurons in response to light-independent oscillating magnetic fields suggests that magnetic information from the trigeminal and vestibular nerves might be processed in the hindbrain (Myklatun et al., 2018). Therefore, it is reasonable to consider that the human hindbrain (e.g., the pons) is also involved in processing light-independent magnetic information. However, identifying specific sensory organs responsible for light-independent magnetoreception in humans, such as the ears, skin, remains challenging. This mini-review aims to explore the mysteries of light-independent pathways by reviewing the literature on the relationship between iron-based metabolic systems and geomagnetism throughout the evolutionary history of life (Supplementary information 1) and by comparing the magnetite hypothesis with the electromagnetic induction hypothesis.

### Earth, the iron planet

One-third of the Earth’s weight is iron, and the Earth is known not only as a water planet but also as an iron planet (Supplementary information 2). In the magma ocean of early Earth, iron and water were abundantly dissolved, reacting chemically to produce iron oxide and hydrogen. It is believed that the geomagnetic field existed shortly after the Earth’s formation, around 4.2 billion years ago (Tarduno et al., 2023). However, the earliest formation of the magnetic field was likely due to the silicate dynamo effect in iron-rich silicate magma, resulting in a different magnetic field shape than today (Stixrude et al., 2020). Later, during the Archean, the iron dynamo effect of molten iron in the core began generating the magnetic field. By the late Archean (2.7 billion years ago), the geomagnetic field had intensified, allowing Proterozoic magnetotactic bacteria to utilize it. Furthermore, the stabilization of the geomagnetic field led to the explosive diversification of life during the Cambrian period (Carlo et al., 2016), and by 370 million years ago, vertebrates had evolved magnetoreception systems using magnetite.

The ubiquity of iron is essential for life because it can participate in various reactions due to its multiple oxidation states, including redox processes and oxygen transport (Frey and Reed, 2012). In the weakly acidic ocean of early Earth, rich in dissolved carbon dioxide, iron ions were abundant, promoting various prebiotic chemical reactions (Eigen, 1971). Iron–sulfur clusters, remnants of the involvement of iron in reactions such as electron transfer before the emergence of life, are evidence of this (Mielke et al., 2011). The last universal common ancestor (LUCA), the primitive life form from which all life descends, could utilize iron in biological systems, including electron transfer (Weiss et al., 2016, 2018). Subsequently, iron-based metabolic systems were inherited throughout the history of life, making iron essential for many biological processes and driving the evolution of life. Iron, as a catalyst, promoted the diversity of life on Earth, leading to the emergence of MTB that produce magnetite.

### The emergence of MTB

The origin of MTB dates back to the Cretaceous period. Nanoscale magnetite crystals have been found within the nasal cells of salmon, existing as small clusters similar to the process by which bacteria produce magnetite (Bellinger et al., 2022). This magnetic sensor in vertebrates likely derives from the genetic systems of ancient bacteria and has evolved over more than two billion years to function as an intracellular magnetic sensor, adapting to the geomagnetic field. The magnetite crystals in the magnetoreceptive cells of organisms suggest that the production of magnetite by bacteria might share a common evolutionary genetic history.

MTB use magnetite nanoparticles to align themselves along the geomagnetic field, allowing them to quickly move to optimal positions in water based on chemical gradients such as oxygen and dissolved iron. Consequently, it was previously thought that MTB could not survive in environments without chemical gradients (Lin et al., 2017). However, a new MTB recently discovered from deep-sea hydrothermal vents suggests that the common ancestor of MTB might have existed independently of chemical gradients as far back as 3.5 billion years ago (Nakano et al., 2023). The common ancestor of MTB might have possessed primitive magnetosomes that served functions other than magnetotaxis, such as detecting gravity, storing iron, or acting as a battery to supply energy through redox cycles (Simmons and Edwards, 2006). Additionally, these bacteria might have used the biosynthesis of magnetic organelles to reduce the toxicity of reactive oxygen species within cells (Lin et al., 2020). During the Archean era, magnetosomes likely formed as byproducts of iron cycling (Strbak and Dobrota, 2019), leading to the emergence of magnetotaxis. This magnetotaxis, interacting with the geomagnetic field, is hypothesized to have evolved into torque-based magnetite receptors essential for light-independent magnetic sensing circuits. Notably, MTB’s biomineralization capability enables the formation of not only magnetite but also other minerals, including calcium carbonate, which form structures such as shells, teeth, and bones (Liu et al., 2021), and might have contributed to the formation of otoliths in the semicircular canals (Lundberg et al., 2015). However, although MTB could produce magnetic particles within cells, the molecular structure of magnetite-activated ion channels in the cell membrane remains unidentified, presenting a significant challenge for researchers.

### Evolution of trigeminal and vestibular nerve system

**Figure 1:**
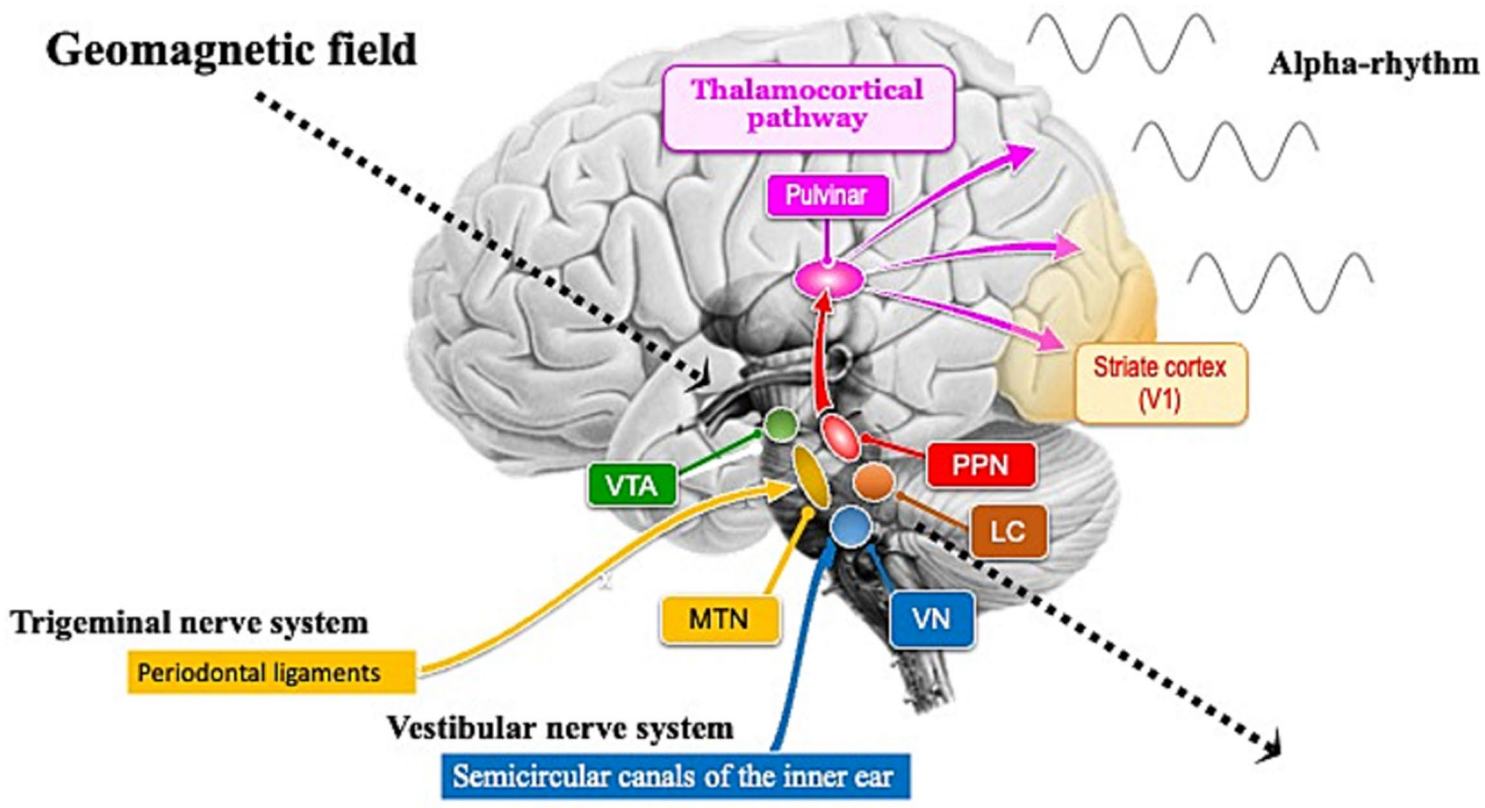
Brain nuclei involved in the generation of alpha rhythms and in the light-independent magnetoreception circuits. The ventral tegmental area (VTA), pedunculopontine nucleus (PPN), and locus coeruleus (LC) are involved in iron metabolism. The VTA functions as a pacemaker for theta rhythms, while the PPN functions as a pacemaker for alpha rhythms. During rest with closed eyes, alpha rhythms are transmitted from the PPN pacemaker to the occipital cortex, particularly the primary visual cortex (V1), through the pulvinar. The LC helps maintain alpha rhythms by promoting wakefulness. Brainstem nuclei involved in light-independent magnetoreception circuits include the mesencephalic trigeminal nucleus (MTN) and the vestibular nuclei (VN). The semicircular canals that send inputs to the VN in the hindbrain were established early in the evolutionary history of the inner ear (Lipovsek and Wingate, 2018; Higuchi et al., 2019). Unlike the highly diverse auditory nuclei across species, the VN is remarkably conserved from fish to humans (Fritzsch and Straka, 2014), suggesting that its slow evolutionary rate indicates its critical functions for survival. One reason for this slow evolutionary rate might be that the VN is not only essential for detecting gravity and head rotation to maintain posture and balance, but also plays a crucial role in processing geomagnetic information. Geomagnetic field (indicated by dotted arrows) steadily reach the brainstem, including the midbrain and hindbrain.

Vertebrates might have evolved a light-independent magnetoreception circuit, possibly derived from the magnetosome gene cluster (Figure 1). In vertebrates such as fish (Walker et al., 1997), birds (Semm and Beason, 1990; Beason and Semm, 1996; Mora et al., 2004; Elbers et al., 2017), and rodents (Wegner et al., 2006), the trigeminal nerve is associated with the magnetoreception circuit. The beaks of birds have been suggested to sense geomagnetic field and involve a magnetoreception circuit through the trigeminal nerve (Cadiou and McNaughton, 2010; Fleissner et al., 2003). Similar magnetoreception circuits might exist in the human trigeminal nerve. Typically, peripheral sensory neurons activated in sensory organs input to the central nervous system through sensory ganglia. Interestingly, the trigeminal mesencephalic nucleus is an exception, as the peripheral sensory neurons themselves are embedded in the brainstem during evolution (Sato, 2021). In other words, proprioceptive sensory information from the periodontal ligament within the oral cavity, which is similar to a bird’s beak, is directly transmitted to the brainstem. Therefore, it is entirely unknown whether the human trigeminal mesencephalic nucleus, in addition to inputting proprioceptive information from the oral cavity, could directly sense geomagnetic field as a magnetoreceptive organ embedded in the brainstem during evolution. This remains a subject for future research.

**Figure 2:**
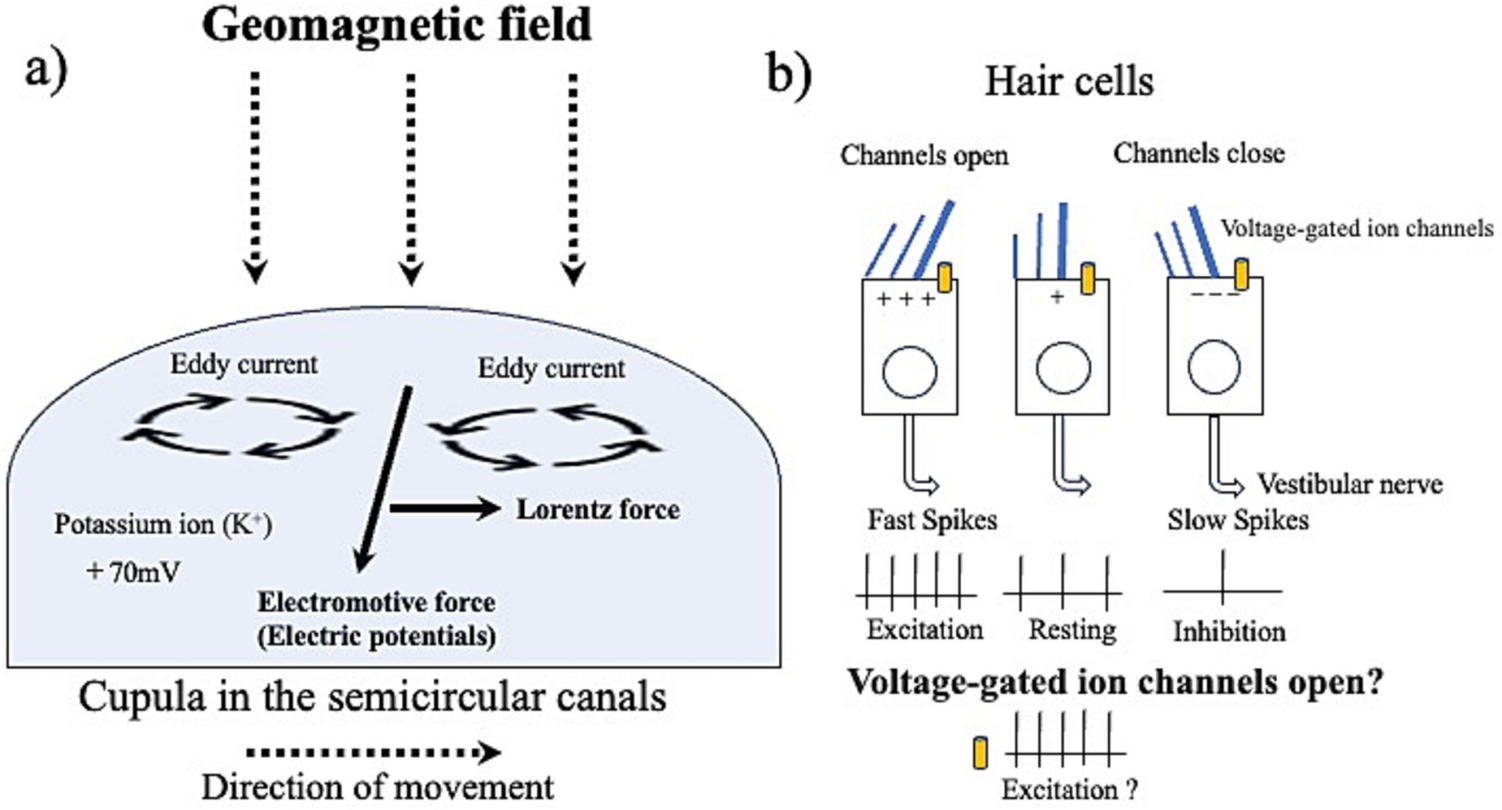
Electromagnetic induction hypothesis. **(A)** Schematic of electromotive force generation in the semicircular canal cupula: based on Arago’s disk principle (Arago and Flourens, 1856), when the semicircular canals move relative to the geomagnetic field, the magnetic flux induces both an electromotive force and a Lorentz force in the cupula, resulting in potential changes. Potassium ions (K+) are actively maintained within the semicircular canal cupula, reaching a potential of +70 mV, creating a unique environment that exceeds the endolymphatic potential of the ampulla (Köppl et al., 2018) and enhances hair cell sensitivity. However, it is still unclear whether the electromotive force induced by geomagnetic field within the conductive cupula could activate hair cells. **(B)** Role of the semicircular canals in geomagnetic field detection: the semicircular canals detect head or body rotation along the vertical, horizontal, and lateral axes through the flow of conductive lymph fluid. Hair cells in the ampulla are covered by a jelly-like structure called the cupula, which tilts hair bundles when lymph fluid flows. This mechanical stimulation is converted into electrical signals and transmitted to the vestibular nerve. Furthermore, since the semicircular canals are arranged along three orthogonal planes, it is suggested that hair cells could activate voltage-gated ion channels by converting relative geomagnetic field movement into potential changes, even if the hair bundles do not tilt (Nimpf et al., 2019).

Magnetotaxis interacting with the geomagnetic field eventually evolved into a specialized sensory organ called the lagena in vertebrates. A hypothesis suggests that magnetized otoliths could be magnetic receptor organs (Harada et al., 2001; Cressey, 2012; Khorevin, 2008). However, recent studies have failed to confirm the presence of magnetite crystals in pigeons’ lagena, indicating that torque-based magnetite receptors in the inner ear are unlikely (Malkemper et al., 2019). Additionally, the lagena, an otolith organ, is present only in fishes, reptiles, and birds, and is absent in mammals, suggesting that the likelihood of torque-based magnetite receptors existing in the human inner ear is low. Instead, a new hypothesis proposes the possibility of voltage-dependent receptors induced by electromagnetic induction through conductive endolymph in the semicircular canals (Nimpf et al., 2019). This hypothesis suggests that, rather than torque-based magnetite receptors, the slight electric potential changes generated in the conductive endolymph are detected by voltage receptors (Figure 2). Therefore, it might be necessary to consider the semicircular canals in humans as candidates for light-independent magnetic reception circuits. To identify light-independent magnetic receptor organs in humans, comprehensive research including peripheral organs (especially the semicircular canals) and specific neural nuclei in the brainstem, which are promising candidates due to their connections with the trigeminal and vestibular systems, might be needed.

### Unique transmission mechanism of geomagnetic field

Humans rely on five basic senses: vision, hearing, touch, smell, and taste. These senses convert stimuli from peripheral organs into electrical signals and transmit information to the central nervous system. But how might humans detect changes in geomagnetic field as a “sixth sense”? Unlike senses such as light and sound, which are shielded from direct transmission to the brain by the skull and cerebrospinal fluid, geomagnetic field uniquely transmit directly to the brain without attenuation from these barriers (Figure 1). The relative permeability of biological tissues is nearly uniform, similar to that of a vacuum, meaning that geomagnetic field transmission is unaffected by variations in tissues like cerebrospinal fluid or the skull. Additionally, for life forms, LUCA, believed to have originated in the deep sea, the magnetic field could propagate almost uniformly through seawater, allowing LUCA to engage in iron-catalyzed metabolism under geomagnetic influence (Supplementary information 3). Following LUCA’s emergence, evolution under geomagnetic influence led to the development of MTB.

Neurons have evolved to coordinate responses to a variety of environmental factors, including molecules, electromagnetic waves, sound waves, and gravity. Gravity, like the geomagnetic field, uniformly affects all cells regardless of their location. Therefore, understanding how cells adapt to gravity is essential for understanding the evolution of magnetic sensors. Plants, for example, use starch statoliths to sense their orientation in response to gravity (Nishimura et al., 2023). Similarly, it is likely that sensory organs capable of detecting subtle changes in geomagnetic field lines exist, prompting researchers to search for magnetized structures within the brain. If these magnetized structures had evolved into large otoconia in the semicircular canals, they would likely have been discovered by now. However, no such magnetized otoconia have been found. Additionally, identifying torque-based, magnetite-activated ion channels within the vast network of brain neurons presents a significant challenge—comparable to finding a needle in a haystack (Johnsen and Lohmann, 2008).

Research suggests that magnetic remanence is higher in the human brainstem and cerebellum than in the cerebral cortex, potentially indicating a greater concentration of magnetite (Gilder et al., 2018). Neuronal melanin in midbrain nuclei such as the ventral tegmental area (VTA), pedunculopontine nucleus (PPN), and locus coeruleus (LC) responds paramagnetically when bound to metals like iron (Sasaki et al., 2006; He et al., 2023). Midbrain nuclei, including the VTA and PPN, exhibit spontaneous rhythmic activity even in the absence of external stimuli, functioning as sources of brainwave activity. The presence of magnetic sensing in neurons near the VTA and PPN could explain brainwave responses to geomagnetic field (Figure 1). However, since the nuclei themselves do not function as geomagnetic sensory organs, the possibility of magnetic sensing in neurons being located near these midbrain nuclei might be considered.

### Magnetic sensing in neurons

Why has not a sensory organ for detecting geomagnetic field been found in humans? The human ear detects changes in gravity using otoconia in the utricle and transmits this information to the vestibular nerve. However, since no magnetized otoconia have been discovered, it is unlikely that human semicircular canals can sense geomagnetic field through magnetized otoconia. Additionally, the trigeminal nerve, which governs the sensory functions of the facial skin, oral mucosa, teeth, and gums, contains mechanoreceptors that respond to pressure, vibration, and skin stretching. These mechanoreceptors are not designed to function as magnetic sensors. Thus, it is challenging to conceive of a physiological mechanism by which humans sense geomagnetic field through the vestibular system of the ear or the trigeminal system of the skin and mucosa.

Therefore, it is necessary to investigate the potential magnetosensitivity of neurons as the remaining candidates for magnetic sensing. The magnetic sensitivity of neurons might be higher than previously imagined. Although distinct from static geomagnetic field, a very weak alternating magnetic field of 10 μT—equivalent to a quarter of the geomagnetic field—has been reported to activate mitochondria within neurons (Tachibana et al., 2024). Mitochondria, which contain large amounts of iron and could sense changes in iron concentration (Bashir et al., 2011), might be sensitive to alternating magnetic fields. Furthermore, astroglia are known to generate very weak biomagnetic fields, which might have promoted self-organization processes and efficient network structures (Martinez-Banaclocha, 2020). These findings suggest that neurons themselves might have evolved to adapt to weak magnetic fields similar to the geomagnetic field. However, since much of the research on magnetic fields uses alternating magnetic fields, it is necessary to consider different physiological mechanisms from those of static geomagnetic field. In the case of alternating magnetic fields, nanoscale magnetite crystals undergo intense rotational motion, making it impossible to sense the direction of the geomagnetic field. On the other hand, in the case of static geomagnetic field, the magnetite crystals maintain a constant tilt, allowing for the detection of the geomagnetic field’s direction.

The PPN, located in the dorsal tegmentum of the brainstem, is highly conserved across species and plays a role in controlling various functions such as movement, reward, motivation, arousal, and behavioral states (Benarroch, 2013). PPN neurons in the brainstem of vertebrates spontaneously and rhythmically fire at an average rate of about 10 Hz (Takakusaki and Kitai, 1997). This PPN functions as the source of the mammalian alpha rhythm (10 Hz) pacemaker and might have enhanced introverted cognitive functions to help nocturnal mammalian ancestors adapt to dark environments (Shibata et al., 2024). The fact that the alpha rhythm of some modern humans responds to magnetic fluctuations in the dark (Wang et al., 2019) suggests that the PPN of nocturnal mammalian ancestors might have been more sensitive to geomagnetic field than modern humans. To determine how the PPN, as a pacemaker of the alpha rhythm, responds to geomagnetic field, it is necessary to identify sensory organs capable of detecting this field (Figure 1). Since neurons might have adaptively evolved to rhythmic electromagnetic fields within the brain, they might be highly sensitive to alternating magnetic fields. Conversely, it might be difficult for them to sense the direction of static geomagnetic field. Therefore, specific sensory organs, such as the semicircular canals, might be required to sense the direction of geomagnetic field.

### Future research

LUCA emerged around 4.2 billion years ago, approximately 300 million years after Earth’s formation, utilizing an iron-based metabolic system (Moody et al., 2024). The geomagnetic field not only influenced iron-based metabolic processes within cells but also aligned countless magnetic particles drifting in the ocean, imposing order on random molecular motion and leading to the magnetization of seabed sediments as detrital remanent magnetization (Verosub, 1989). In the ancient oceans, dissolved magnetic particles might have repeatedly attached to and detached from cell membranes. Through the influence of the geomagnetic field, the interaction between randomly floating magnetic particles outside the cell and the ordered magnetic particles within the cell might have created an environment conducive to detecting the geomagnetic field. As a result, MTB might have evolved the ability to produce magnetite as magnetic sensor.

Around 600 million years ago, an ancestor of neurons began transmitting signals between cells through neuropeptides (Najle et al., 2023). These neuropeptides might have been enclosed and exocytosed by membranes rich in iron-containing phospholipids. Consequently, neuronal cell membranes were highly vulnerable to the accumulation of iron-dependent lipid peroxides, making them prone to ferroptosis, a form of cell death dependent on iron (Jacquemyn et al., 2024). However, even if iron-dependent cell membranes increased sensitivity to geomagnetic field, significant challenges remain in discovering magnetite-activated, torque-based ion channel that use magnetite for opening and closing, as the molecular structure of these receptors is unknown. Furthermore, with no observed magnetic monopoles and the difficulty organisms face in utilizing magnetically charged particles (Bai et al., 2021), neurons in nature might have evolved voltage-dependent channels using positively and negatively charged particles instead. Sharks, in particular, evolved the Lorenzini ampullae, an ultra-sensitive electroreceptive system that detects weak electric potential differences between fish and seawater (Kalmijn, 1971). Therefore, the brain might have adopted an evolutionary strategy to convert geomagnetic field into electric potentials, utilizing voltage-dependent channels (Figure 2). The unique structure of the semicircular canals, with three semicircular tubes orthogonal to each other, is particularly suited to separate geomagnetic field into force (such as gravity and rotational motion) and electric potentials according to Fleming’s left-hand rule (Nimpf et al., 2019). This paper introduces both the magnetite and electromagnetic induction hypotheses, which need not be mutually exclusive. It is plausible that neurons might use magnetite to enhance magnetic sensitivity as an adaptation to evolving brainwave environment, while the semicircular canals might utilize electromagnetic induction to detect the orientation of geomagnetic field. Future research should explore both the magnetite hypothesis, suggesting direct sensitivity to geomagnetic field, and the electromagnetic induction hypothesis, which posits that semicircular canals convert geomagnetic field into electric potentials.

## Conclusion

Eukaryotic cells, and vertebrates have developed magnetoreception systems to adapt to the geomagnetic field. Numerous studies on magnetoreception in birds, particularly concerning the upper beak and inner ear, suggest that in humans, the trigeminal nerve, vestibular nerve, and hindbrain might be involved in light-independent magnetoreception pathways. However, the specific sensory organ in humans that detects the geomagnetic field has not yet been identified. Unlike traditional senses, geomagnetic information is transmitted without attenuation through the scalp, bones, and cerebrospinal fluid, similar to gravity. It also generates eddy currents and lorentz forces accompanying the relative movement of the geomagnetic field (Arago and Flourens, 1856). Considering these unique properties, vertebrates might have evolved to detect the geomagnetic field in a light-independent manner, not only through direct detection using torque-based magnetic particles but also through indirect detection of electric potentials using electromagnetic induction. Further research into this evolutionary adaptation could help unravel the mystery of geomagnetic field detection in humans.
