# Supplementary material for: The origins of light-independent magnetoreception in humans
Source: Front Hum Neurosci. 2024 Nov 29;18:1482872. doi: 10.3389/fnhum.2024.1482872 (PMC11638171; doi:10.3389/fnhum.2024.1482872)
Supplement: Supplementary file 1 [file Data_Sheet_1.docx]

Supplementary Material

**The Origins of Light-Independent Magnetoreception in Humans**

**Takashi Shibata ^1,2^** ***, Noriaki Hattori ^3^, Hisao Nishijo^4^, Satoshi Kuroda ^1^, and Kaoru Takakusaki ^5^**

^１^Department of Neurosurgery, Toyama University Hospital, Japan

^2^ Department of Neurosurgery, Toyama Nishi General Hospital, Japan

^3^ Department of Rehabilitation, Toyama University Hospital, Toyama, Japan

^4^ Faculty of Human Sciences, University of East Asia, Yamaguchi, Japan

^5^ The Research Center for Brain Function and Medical Engineering, Asahikawa Medical University, Asahikawa, Japan

# Corresponding Author: Takashi Shibata, MD, PhD, E-mail:sibata@dj8.so-net.ne.jp

1. **
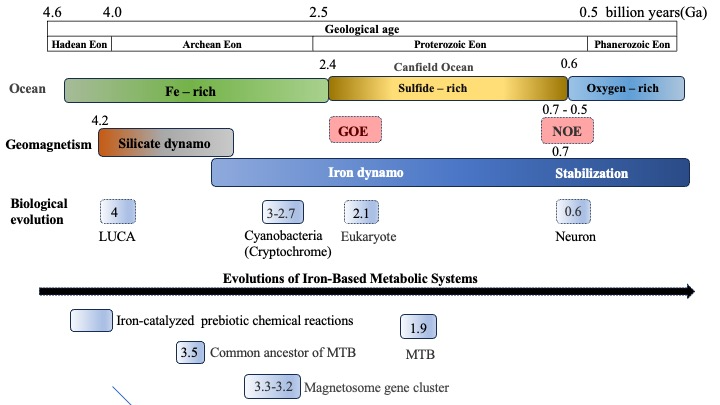
**

**Supplementary information.1**

The relationship between biological evolution, the oceans (Canfield and Teske, 1996, Canfield, 1998; Toyokuni et al, 2023), and geomagnetism (Tarduno et al, 2023; Stixrude et al, 2020) has been summarized according to geological eras. After the Great Oxidation Event (GOE), the ancient iron-rich oceans underwent significant changes, transitioning over approximately a billion years from the low-oxygen, sulfur-rich Canfield Ocean to the oxygen-rich modern oceans. The GOE triggered a metabolic revolution, shifting from anaerobic to aerobic metabolism, and also led to the emergence of neurons—cells that consume large amounts of oxygen—which originated from peptidergic cells around 600 million years ago (Najle et al., 2023). To meet these high oxygen demands, neurons developed mechanisms to prevent iron-dependent cell death while simultaneously evolving an iron-catalyzed mitochondrial electron transport chain, promoting the neoteny of human-specific neurons (Casimir et al., 2024). The evolution of iron-based metabolic systems, driven by the emergence of the magnetosome gene cluster (Lin et al.,2017) and magnetotactic bacteria (Kopp and Kirschvink., 2008), led to neuron cell membranes becoming rich in iron-containing phospholipids, potentially increasing their sensitivity to magnetoreception. The stabilization of the geomagnetic field and Neoproterozoic Oxygenation Event (NOE) (Lee et al., 2016) around 700 million years ago coincides with the emergence of neurons, suggesting that not only abundant oxygen but also the stabilization of the geomagnetic field might have been crucial to the birth of neurons. Abbreviations: GOE: Great Oxidation Event, NOE: Neoproterozoic Oxygenation Event, LUCA: Last Universal Common Ancestor. MTB: Magnetotactic Bacteria. The numerical values represent geological eras in billions of years (Ga).

**Supplementary information.2**


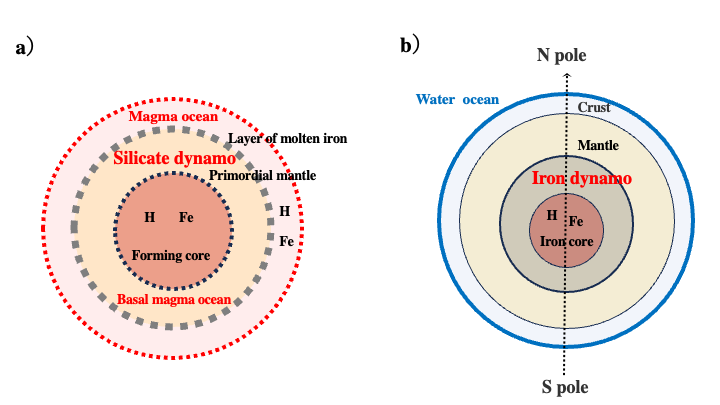


   Paleomagnetism from the Archean eon (about 4 billion to 2.5 billion years ago) indicates that the early Earth had a magnetic shield different from the present-day magnetic field (Stixrude et al, 2020). This paleomagnetic field, caused by a silicate dynamo, provided unique protection for the primordial Life. Furthermore, during the formation of the Earth, a significant amount of water was absorbed into the Earth's core as hydrogen, while iron oxides dissolved into the magma ocean (Nomura et al, 2014). Therefore, the Last Universal Common Ancestor (LUCA) lived in an environment where iron oxides were available. The primitive Earth, composed of primordial materials, remained undifferentiated, with dashed lines indicating that the crust, mantle, and core were not distinctly separated. b) Geomagnetism from about 2.5 billion years ago to the present, generated by the iron dynamo. Many organisms have evolved under stable geomagnetic field.

**Supplementary information.3**

**
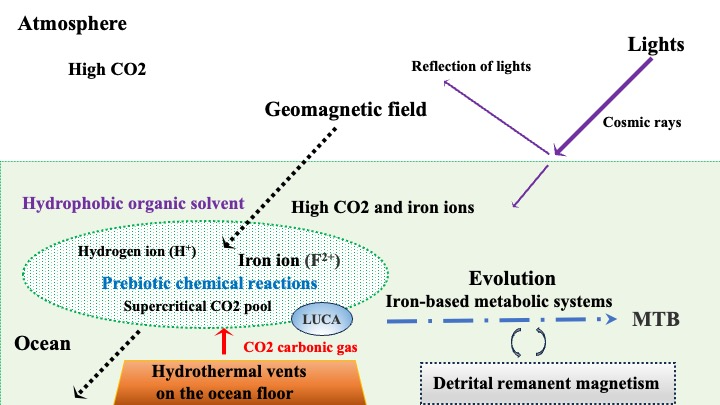
**

**The Ancient Oceans**

   The supercritical carbon dioxide liquid discovered at hydrothermal vents on the seafloor has unique chemical properties as a hydrophobic organic solvent, which repels water and might have served as a cradle for early life (Shibuya and Takai, 2022). In this unique environment, prebiotic chemical reactions—such as the bonding of organic molecules catalyzed by iron—were abundant and could have contributed to the emergence of the last universal common ancestor (LUCA). Additionally, while the sea surface reflected harmful light, the geomagnetic field (a dotted arrow) consistently reached the seafloor. This iron-based metabolic system might have interacted with magnetic particles drifting through the ocean along the direction of the geomagnetic field (Detrital remanent magnetism) (Verosub, 1989), potentially leading to the emergence of magnetotactic bacteria (MTB). The ancient oceans might have appeared light green due to the high concentration of dissolved ferrous iron ions (Fe²⁺). Abbreviations: RNA: Ribonucleic acid, CO2: Carbon dioxide.

**References**

Arago, Francois; Flourens, Pierre, “Ceuvres Completes De Francois Arago,” Paris: Gide et J. Baudry. vol.4. pp. 424. 1856.

Canfield, D. E., Teske, A. (1996). Late Proterozoic rise in atmospheric oxygen concentration inferred from phylogenetic and sulphur-isotope studies. Nature, 382(6587), 127–132. https://doi.org/10.1038/382127a0

Canfield, D. (1998). A new model for Proterozoic ocean chemistry. Nature 396, 450–453. <https://doi.org/10.1038/24839>

Casimir, P., Iwata, R., Vanderhaeghen, P. (2024). Linking mitochondria metabolism, developmental timing, and human brain evolution. Current opinion in genetics & development, 86, 102182. <https://doi.org/10.1016/j.gde.2024.102182>

Kopp, R. E. Kirschvink, J. L. (2008). The identification and biogeochemical interpretation of fossil magnetotactic bacteria. Earth-Sci. Rev.86, 42–61.

Lin, W., Paterson, G. A., Zhu, Q., Wang, Y., Kopylova, E., Li, Y., et al. (2017). Origin of microbial biomineralization and magnetotaxis during the Archean. *Proceedings of the National Academy of Sciences of the United States of America*, *114*(9), 2171–2176. 

Lee, CT., Yeung, L., McKenzie, N. et al. (2016). Two-step rise of atmospheric oxygen linked to the growth of continents. Nature Geosci 9, 417–424. <https://doi.org/10.1038/ngeo2707>

Najle, S. R., Grau-Bové, X., Elek, A., Navarrete, C., Cianferoni, D., Chiva, C., et al. (2023). Stepwise emergence of the neuronal gene expression program in early animal evolution. Cell, 186(21), 4676–4693.e29. <https://doi.org/10.1016/j.cell.2023.08.027>

Najle, S. R., Grau-Bové, X., Elek, A., Navarrete, C., Cianferoni, D., Chiva, C., et al. (2023). Stepwise emergence of the neuronal gene expression program in early animal evolution. Cell, 186(21), 4676-4693.e29. <https://doi.org/10.1016/j.cell.2023.08.027>

Nimpf, S., Nordmann, G. C., Kagerbauer, D., Malkemper, E. P., Landler, L., Papadaki-Anastasopoulou, A., et al. (2019). A Putative Mechanism for Magnetoreception by Electromagnetic Induction in the Pigeon Inner Ear. Current biology : CB, 29(23), 4052–4059.e4. <https://doi.org/10.1016/j.cub.2019.09.048>

Nomura, R., Hirose, K., Uesugi, K., Ohishi, Y., Tsuchiyama, A., Miyake, A., & Ueno, Y. (2014). Low core-mantle boundary temperature inferred from the solidus of pyrolite. Science (New York, N.Y.), 343(6170), 522–525. <https://doi.org/10.1126/science.1248186>

Stixrude, L., Scipioni, R., Desjarlais, M. P. (2020). A silicate dynamo in the early Earth. Nature communications, 11(1), 935. <https://doi.org/10.1038/s41467-020-14773-4>

 Shibuya, T., Takai, K. (2022). Liquid and supercritical CO2 as an organic solvent in Hadean seafloor hydrothermal systems: implications for prebiotic chemical evolution. Prog Earth Planet Sci 9, 60. <https://doi.org/10.1186/s40645-022-00510-6>

Tarduno, J. A., Cottrell, R. D., Bono, R. K., Rayner, N., Davis, W. J., Zhou, T., et al. (2023). Hadaean to Palaeoarchaean stagnant-lid tectonics revealed by zircon magnetism. Nature, 618(7965), 531–536. <https://doi.org/10.1038/s41586-023-06024-5>

Toyokuni, S., Kong, Y., Zheng, H., Maeda, Y., Katabuchi, M., Motooka, Y. (2023). Three-Dimensional Regulation of Ferroptosis at the Intersection of Iron, Sulfur, and Oxygen Executing Scrap and Build Toward Evolution. Antioxidants & redox signaling, 39(10-12), 807–815. <https://doi.org/10.1089/ars.2022.0142>

Verosub, K.L. (1989). Detrital remanent magnetism (DRM) .In: Geophysics. Encyclopedia of Earth Science. Springer, Boston, MA. https://doi.org/10.1007/0-387-30752-4_25
